# A Broadband Switched-Beam Antenna with Angle-of-Arrival Estimation Capability

**DOI:** 10.3390/s26123760

**Published:** 2026-06-12

**Authors:** Jeen-Sheen Row, Yu-Jie Lin

**Affiliations:** Department of Electrical Engineering, National Changhua University of Education, Chang-Hua 500, Taiwan

**Keywords:** reconfigurable antenna, circular array, wideband operation, angle-of-arrival estimation

## Abstract

This paper presents a wideband pattern-reconfigurable antenna designed for 360° horizontal sensing with angle-of-arrival (AoA) estimation capability. The antenna features a unique three-layer planar architecture, where a microstrip circular array is integrated between two metallic plates to enhance radiation stability and bandwidth. By employing a single-pole four-throw (SP4T) switching circuit, the array generates four steerable beams covering the entire azimuthal plane. Experimental results show that the prototype achieves a 10 dB return loss impedance bandwidth of 50% (4.0–6.0 GHz) and a peak gain of 8.3 dBi. Based on this antenna, a correlation-coefficient-based AoA estimation approach is implemented. The measured results demonstrate reliable estimation performance, with a mean angular error of less than 1.5° over the 360° horizontal plane across the operating frequency range. The proposed design provides a compact and low-complexity solution for practical wideband direction-finding applications in next-generation wireless systems.

## 1. Introduction

With the rapid evolution of next-generation wireless communication and the conceptualization of 6G technology, the demand for multifunctional antenna systems has reached unprecedented levels. Modern wireless infrastructures, particularly Integrated Sensing and Communication (ISAC) frameworks, require antenna that can provide both high-capacity data transmission and precise spatial sensing capabilities. Among various spatial sensing techniques, angle-of-arrival (AoA) estimation has become a fundamental enabler for diverse applications, ranging from indoor positioning and asset tracking to autonomous vehicle navigation (V2X) and unmanned aerial vehicle (UAV) coordination. Generally, AoA estimation techniques can be categorized according to their signal-processing principles and implementation complexity. The first category involves high-resolution subspace-based algorithms, such as Multiple Signal Classification (MUSIC) [1] and Estimation of Signal Parameters via Rotational Invariance Techniques (ESPRIT) [2], which provide superior estimation accuracy but usually require multi-channel RF front-ends and intensive matrix operations. The second category focuses on low-complexity direction-finding approaches, including recent studies on one-bit AoA estimation techniques [3,4,5], which reduce hardware complexity and computational cost for practical implementations. Furthermore, recent developments in adaptive and pattern-reconfigurable beam-steering architectures, including DDS-PLL-based adaptive beam-forming systems, have demonstrated the feasibility of practical real-time directional wireless systems [6]. Such systems commonly employ phased-array or dynamically reconfigurable antenna structures to realize agile beam control and adaptive wireless coverage. Although these approaches provide high beam-steering flexibility and directional accuracy, they generally involve increased RF front-end complexity, active phase-control circuits, and higher power consumption. In contrast, the switched-beam antenna approach investigated in this work offers a simple and cost-effective solution for low-complexity IoT-oriented direction-finding and localization applications, including switched-beam-based indoor localization scenarios [7].

To achieve accurate AoA estimation over a wide spatial range, antenna systems must be capable of scanning or switching beams across the azimuthal plane. A considerable number of beam-steerable antenna designs have been proposed for decades. Some of the reported designs are fabricated on a single substrate for the advantages of compact size and low profile [8,9,10,11,12,13,14]. For example, the antenna in [8] realizes four-beam switching within an area of 0.36 λ_0_ × 0.36 λ_0_, but the available bandwidth is only 8%. To improve bandwidth, the design in [9] uses an arc dipole fed with a balun as the element of a circular array, and the impedance bandwidth is considerably increased to 33.6%; however, the maximum gain of the resultant beam is only 4.1 dBi across the operation frequencies. In [10], a planar reconfigurable antenna based on a Yagi–Uda structure has been proposed to obtain a steerable beam with a peak gain of 7.3 dBi at the cost of increasing the antenna area by a factor of nine, as compared to the design in [9]; on the other hand, the obtained impedance bandwidth is decreased to 12%. A wideband slot antenna has also been adopted to realize beam scanning [14], and it can give four beams with the maximum gain of 6.4 dBi within 70% impedance bandwidth. However, the radiation patterns of the switched beams are not stable against frequency, and their side lobe levels (SLLs) are up to −3 dB, leading to a high correlation among the switched beams.

To realize a pattern-reconfigurable antenna with high gain and stable beams, several designs based on a three-dimensional (3D) structure have been reported [15,16,17,18,19,20,21]. The design in [16] uses a nonagon shape with a switchable frequency selective surface surrounding a radiating source, and the switched beam has a peak gain of 8.6 dBi. The main drawback of the design is that the impedance bandwidth is only 8.5%. The similar gain can be achieved using a planar circular array surrounded by a 3D-printed dielectric lens radome [17], and the impedance bandwidth is improved to 18.2%; however, the radiation pattern of the directional beam at higher operation frequencies has a SLL of −5 dB, which needs to be improved for the case that the beam is steered in the whole horizontal plane. The gain of the scanned beams can be further enhanced to around 10 dBi when ring-shaped reflectors [18] or bowl-shaped reflectors [19] are introduced in antenna designs, and these two designs can provide 14.5% and 26.2% impedance bandwidths, respectively. Nevertheless, such a reflector with a curved surface is relatively difficult to implement compared to a flat metallic plate.

In this paper, a design for a wideband pattern-reconfigurable circular array integrated with a single-pole four-throw (SP4T) switching circuit is proposed. The proposed antenna architecture features a novel three-layer planar configuration: a microstrip circular array is positioned in the middle layer and sandwiched between two metallic plates. This sandwich structure is strategically designed to enhance antenna gains within an impedance bandwidth of approximately 50%. Compared with conventional switched-beam antennas, the proposed design simultaneously achieves wide impedance bandwidth, stable beam characteristics, low side lobe levels, and practical AoA estimation capability over the full 360° azimuth plane using a simple low-complexity architecture. The remainder of this paper is organized as follows. Section 2 describes the geometry of the circular array and the detailed design. Section 3 provides the simulation analysis of the sandwich structure. Section 4 presents the experimental results of the fabricated prototype. Section 5 discusses the AoA estimation algorithm and system-level validation results, followed by conclusions in Section 6.

## 2. Circular Array and Analyses

**Figure 1:**
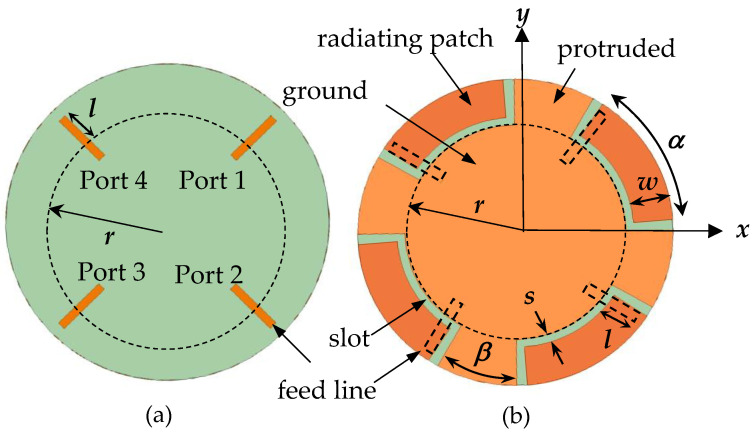
Layouts of the proposed circular array fabricated on FR4 substrate; (**a**) top side; (**b**) bottom side.

Figure 1a,b depict the layouts of top and bottom sides of a circular microstrip array fabricated on an FR4 substrate of thickness 0.76 mm and relative permittivity 4.4. The array has four elements, and each element has an arc-shaped radiating patch of width *w* and central angle *α*. A circular patch with a radius of *r* and four protruded patches with central angle *β* serve as the ground plane. The arc-shaped radiating patch and the circular ground plane have a gap of *s*, and they are both printed on the bottom side. Consequently, the array element can be regarded as a coplanar microstrip antenna whose operation frequency is mainly determined by *r*, *s*, and *α*. A 50 Ω feed line fabricated on the top side is used to excite each element through coupling, and the impedance matching can be obtained by tuning the stub length *l*.

**Figure 2:**
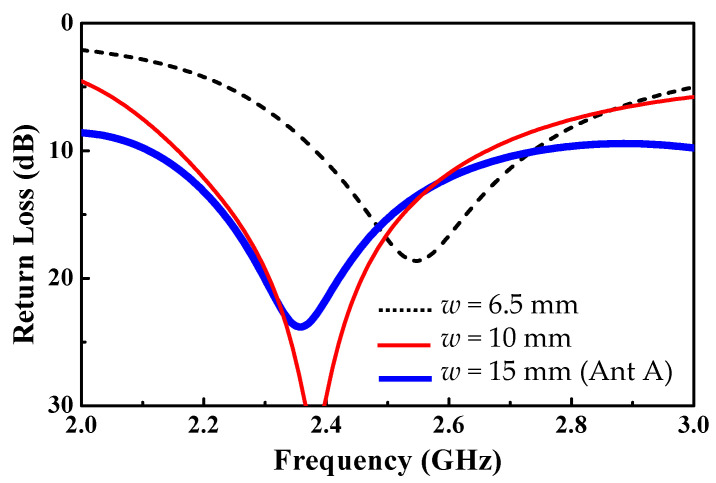
Effects of varying *w* on return loss for Example Ant: *r* = 28 mm, α = 52°, β = 15°, *s* = 2 mm, and *l* = 6.5 mm.

An example (Example Ant) with the dimensions of *r* = 28 mm, α = 52°, β = 15°, *w* = 6.5 mm, *s* = 2 mm, and *l* = 6.5 mm is selected to show the performances of the array element. Figure 2 presents the HFSS-simulated return loss at Port 1, and the results indicate that the resonant frequency, defined by the minimum return loss, of the radiating element is around 2.54 GHz. As the other parameters are fixed, the effects of varying *w* are also outlined in Figure 2. From the results, it can be observed that with increasing *w*, the variations in the return loss against frequency are significantly reduced, while the resonant frequency only has a decrease of 5%. The consequence is that the 10 dB return loss impedance bandwidth is improved from 14% to 27% when *w* is widened from 6.5 to 15 mm.

**Figure 3:**
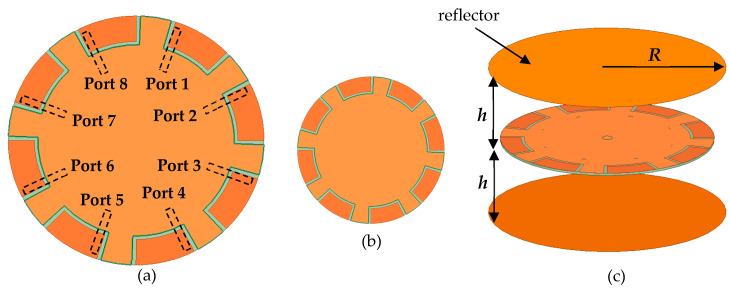
Layouts of the bottom sides for Ant B and C; other dimensions are the same as those of Example Ant. (**a**) Ant B: *r* = 56 mm, α = 26°, *w* = 15 mm, *l* = 13 mm, and *s* = 2 mm; (**b**) Ant C: *r* = 28 mm, α = 26°, *w* = 7.5 mm, *l* = 6.5 mm, and *s* = 1 mm; and (**c**) Ant C with dual reflectors: *h* = 25 mm and *R* = 38 mm.

**Figure 4:**
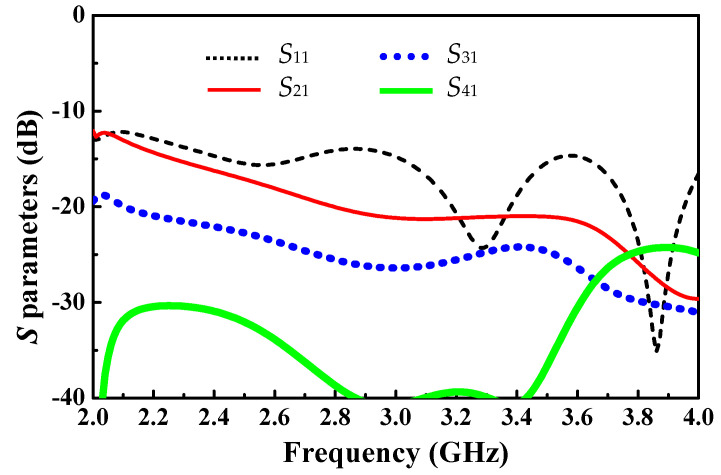
Frequency responses of *S* parameters for Ant B.

**Figure 5:**
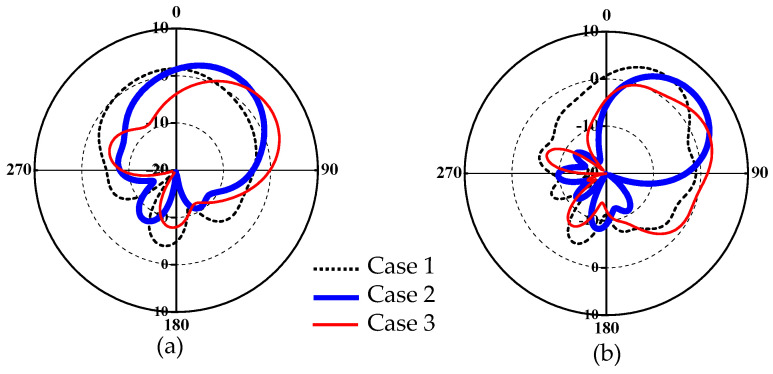
Co-pol (*E*_ϕ_) radiation patterns in *x–y* plane for Ant B operated in Cases 1, 2, and 3; cross pol (*E*_θ_) is smaller than −20 dB: (**a**) 2 GHz; (**b**) 3 GHz.

For the *w* = 15 mm case (Ant A) studied in Figure 2, the number of the array element can be increased by a factor of 2 provided that the parameter *r* is doubled while at the same time *α* is reduced by half, and the resultant circular array is named Ant B, as shown in Figure 3a. It has to be noted that Ant A and Ant B have the same dimensions except *r*, *α*, and *l*. Figure 4 presents the simulated *S* parameters of Ant B. The results demonstrate that the impedance bandwidth of the array element is more than 60%, and the isolation level between the array elements is lower than −12 dB within the bandwidth. The radiation patterns of Ant B are explored for the three cases including one port excited (Case 1), two adjacent ports excited (Case 2), and three adjacent ports excited (Case 3). The results simulated at 2 and 3 GHz are illustrated in Figure 5, and they clearly prove that Case 2 not only realizes a higher gain but also has lower back lobes at each frequency than the other two cases.

**Figure 6:**
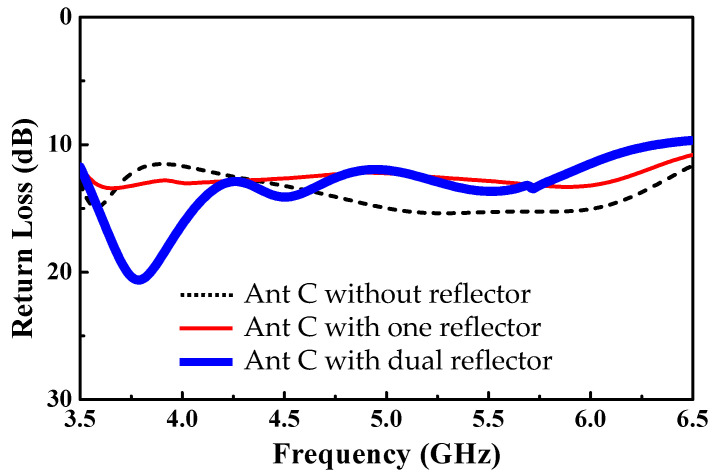
Return loss results of Ant C with and without reflector.

**Figure 7:**
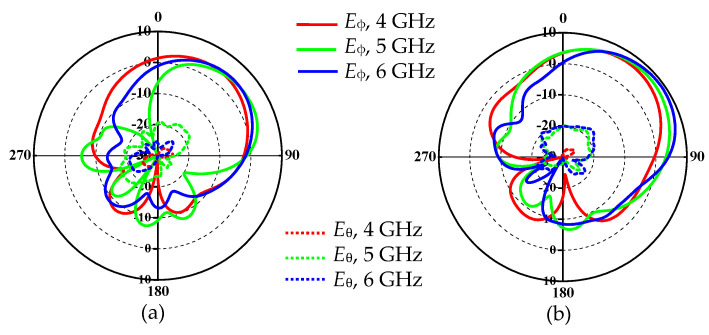
Radiation patterns in *x–y* plane for Ant C at different frequencies: (**a**) without reflector; (**b**) with dual reflectors.

**Figure 8:**
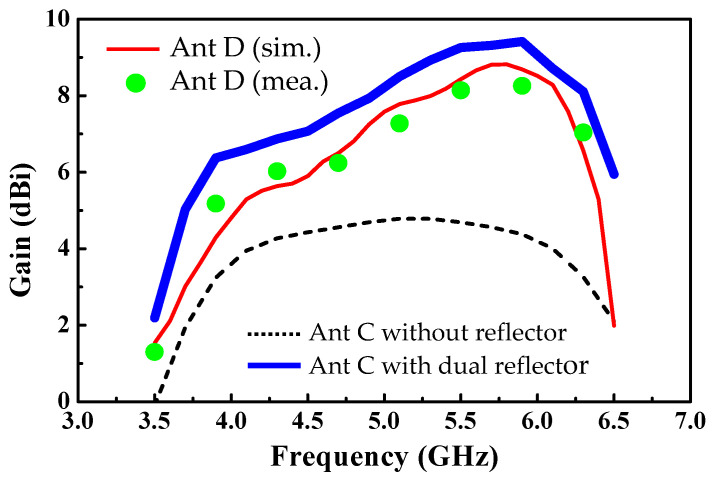
Gain variations in Ant C and Ant D against frequency.

The operation band of Ant B can be easily moved to other frequencies as long as the parameters (*r*, *s*, *w*, *l*) are scaled with a factor of *k*. For the case of *k* = 0.5, the element layout of the resultant array (Ant C) is exhibited in Figure 3b. For Ant C, the simulated data is shown in Figure 6, and a wide impedance bandwidth centered at 5 GHz is seen as expected. Figure 7a plots the radiation patterns of Ant C at 4, 5, and 6 GHz when two adjacent ports (Ports 1 and 2) are simultaneously excited, namely in Case 2. Stable beams directed to ϕ = 45° are obtained. Figure 8 presents the gain variations in the beam against frequency. The gain is 4.4 ± 0.4 dBi in the frequency range from 4 to 6 GHz. For the studied antennas mentioned above, example antenna operating at 2–3 GHz is first investigated to verify the fundamental switched-beam operating mechanism. The antenna dimensions are subsequently scaled to realize Ant B operating in the 4–6 GHz band, and the number of array elements is further increased in Ant C to improve beam-switching characteristics.

## 3. Circular Array with Reflectors

**Figure 9:**
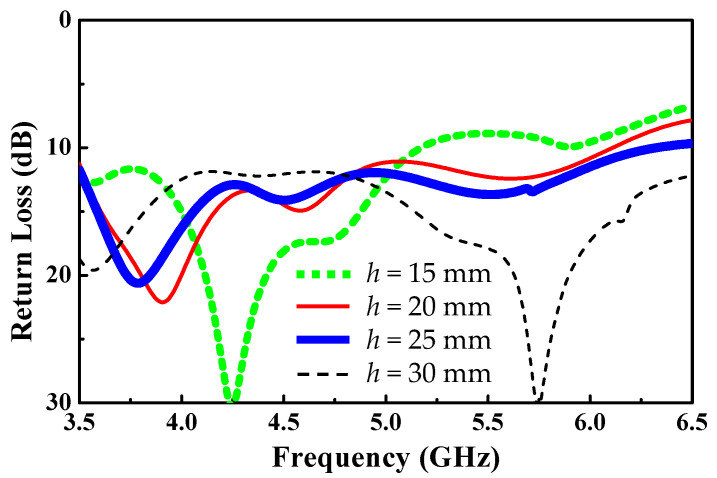
Effects of varying *h* on return loss of Ant C with dual reflectors.

The gain of Ant C can be increased by introducing a pair of reflectors, as depicted in Figure 3c. The two reflectors are circular metallic plates with a radius (*R*) of 38 mm, and these are placed above and below the microstrip circular array at a distance (*h*) of 25 mm. Consequently, the total size of the formed antenna is about 1.3 λ_0_ × 1.3 λ_0_ × 0.85 λ_0_, where λ_0_ is the free-space wavelength corresponding to 5 GHz. Figure 6 shows the return loss of the array element when the dual reflectors are introduced into Ant C. The results are also given for the case where only the top reflector is added. From the simulated results, it can be seen that the effects of the reflectors on impedance bandwidth are not obvious. It is worth noting that a return loss smaller than −10 dB can be achieved from 3.5 to 6 GHz as long as *h* is larger than 20 mm, as shown in Figure 9. When Port 1 and Port 2 are simultaneously activated, the radiation patterns of Ant C with the two reflectors are plotted in Figure 7b. For each frequency, a directive beam with a front-to-back ratio of more than 15 dB is found in the *x*–*y* plane, and the cross-polarization level is lower than −25 dB; moreover, no side lobes are observed. The gains of the beams at different frequencies are provided in Figure 8. The maximum gain occurs at 5.9 GHz, and it is up to 9.5 dBi. Within the frequency band from 4 to 6 GHz, the average gain is 8 dBi, which is 3.6 dBi higher than that of Ant C without any reflector. The increased gain is due to the fact that the two metallic plates act as a boundary to suppress the θ-plane radiation, effectively focusing the electromagnetic energy toward the ϕ-plane. It can be expected that the scanned beams, pointed to ϕ = 45° (Mode 1), 135° (Mode 2), 225° (Mode 3), and 315° (Mode 4), can be carried out when the circular array is integrated with four Wilkinson power dividers and a SP4T circuit. The improved directivity and reduced beam overlap of Ant C also enhance the distinguishability between adjacent switched beams, thereby improving the angular discrimination capability for AoA estimation.

## 4. Experimental Results of the Constructed Prototype

**Figure 10:**
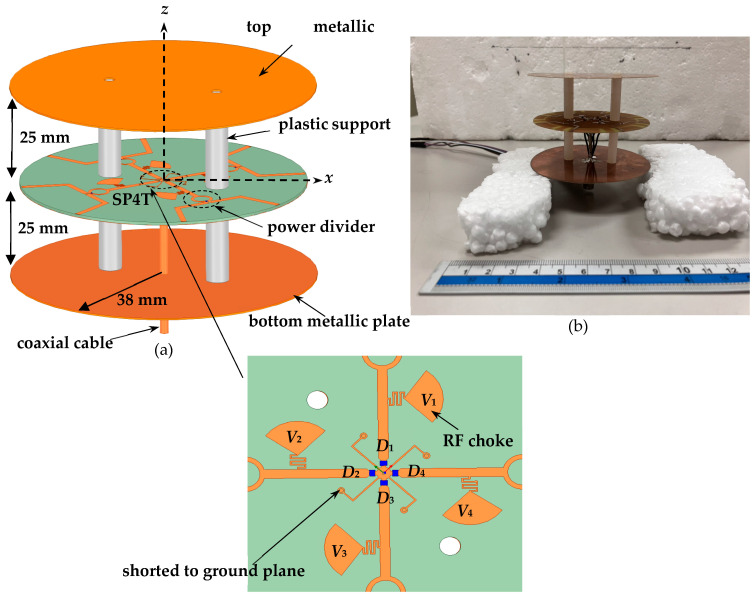
The structure of the proposed pattern-reconfigurable antenna. (**a**) The whole structure of the reconfigurable antenna (Ant D); (**b**) photograph of the constructed prototype.

**Figure 11:**
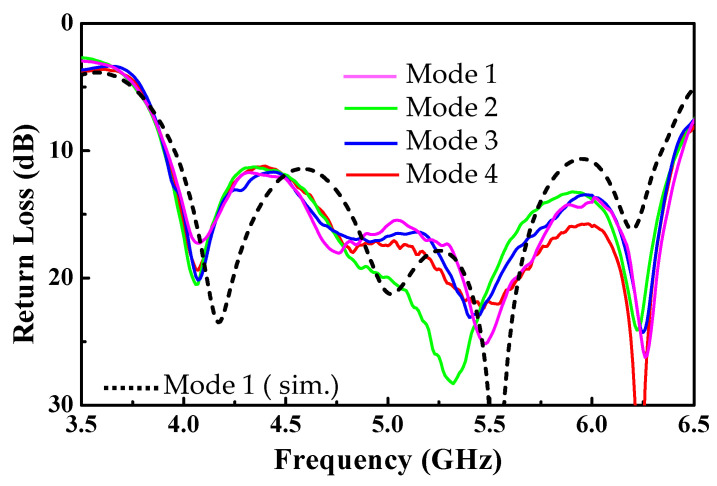
Measured return loss of Ant D operated in different modes, along with the simulated results of Mode 1.

**Figure 12:**
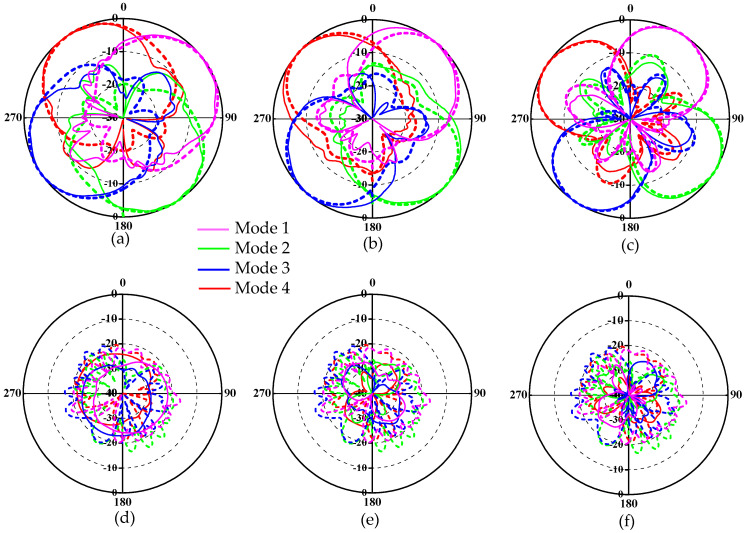
Measured and simulated radiation patterns of Ant D operated in different modes at three frequencies; solid lines are measured data and dashed lines are simulated data. (**a**) *E*_ϕ_ at 4 GHz; (**b**) *E*_ϕ_ at 5 GHz; (**c**) *E*_ϕ_ at 6 GHz; (**d**) *E*_θ_ at 4 GHz; (**e**) *E*_θ_ at 5 GHz; (**f**) *E*_θ_ at 6 GHz.

The whole structure of the proposed pattern-reconfigurable antenna (Ant D) is depicted in Figure 10a, and a photograph of the constructed prototype is presented in Figure 10b. The SP4T circuit comprises four PIN diodes (MA4SPS402) and RF chokes, and each of its outputs are connected to the feed line of the array element through a 1-to-2 Wilkinson power divider. The reconfigurable antenna is center fed with a coaxial cable so that the four radiation modes have the same electrical performances. As (*V*_1_, *V*_2_, *V*_3_, *V*_4_) is set to (1V, 0, 0, 0), (0, 1V, 0, 0), (0, 0, 1V, 0), and (0, 0, 0, 1V), the operation modes of the prototype correspond to Mode 1, Mode 2, Mode 3, and Mode 4, respectively. Figure 11 shows the measured return loss at the input port of the cable when the antenna is operated in Modes 1, 2, 3, and 4. The simulated results of Mode 1 are also given for comparison. In the simulation, the diodes at ON and OFF states are modeled as a 5 Ω resistor and 0.05 pF capacitor, respectively. In Figure 11, the experimental results of the antenna operating in each mode are similar, and they also have satisfactory agreements with the simulated data. The measured impedance bandwidth is about 50% with respect to the center frequency 5.1 GHz. Figure 12 plots the steerable beams of the pattern-reconfigurable antenna measured at three different frequencies. The four main beams obtained at each test frequency are symmetrically scanned in the whole horizontal plane, and they are also stable within the impedance bandwidth. The half-power beamwidths of the main beams at 4, 5, and 6 GHz are 68°, 60°, and 54°, respectively. Regarding the peak gains of the beams, their simulated and measured results against frequency are presented in Figure 8. According to the measured results, the maximum gain is 8.3 dBi, and the average gain is about 7 dBi across the whole operation frequencies. It has to be mentioned that the gain of Ant D is around 1.2 dB lower than that of Ant C with the reflectors. The errors mainly come from the loss of the Wilkinson power dividers and the diodes, which are not considered in the simulation of Ant C. It suggests that the magnitudes of these ohmic losses determine the antenna efficiency of Ant D. With the simulated directivity of Ant C with reflectors and the measured gain of Ant D, the efficiency of the proposed reconfigurable antenna is evaluated, and it is about 60% within the impedance bandwidth. Table 1 provides the comparisons between the proposed and reported designs for the beam-steerable antennas. The proposed design apparently has a wider impedance bandwidth than the other designs, and it can also achieve a SLL, including back lobes, smaller than −10 dB within such a bandwidth.

## 5. Angle-of-Arrival Estimation System

**Figure 13:**
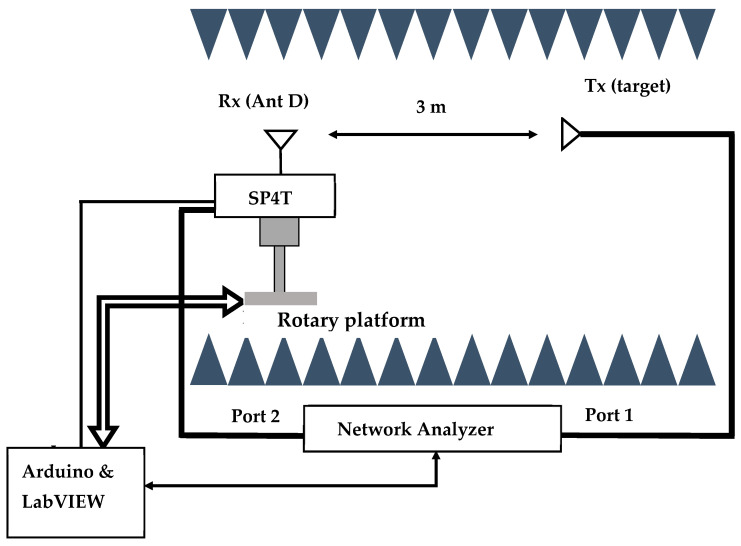
Experimental setup of the angle-of-arrival estimation.

A simple angle-of-arrival system is realized with Ant D, and the experiments are carried out in an anechoic chamber, as shown in Figure 13. Ant D is mounted on a rotary platform as the receiver (Rx), while a reference horn antenna serves as the transmitter (Tx). Guided by a LabVIEW interface, an Arduino microcontroller drives the SP4T circuit to sequentially cycle through the four radiation modes. The vector network analyzer subsequently measures the received power amplitude (*S*_21_) for each mode and routes the data back to LabVIEW.

For Ant D, the measured radiation patterns of the four switched beams in the horizontal plane (θ = 90°) are recorded in advance, and they are expressed as *F*_n_(ϕ). When the signal is transmitted to Ant D in a specific direction (ϕ_A_), controlled by the rotary platform, the four beams of Ant D are sequentially switched, and the corresponding received power is denoted as *Y_n_* (*n* = 1, 2, 3, 4). The correlation coefficient Γ(ϕ) between *F*_n_(ϕ) and *Y*_n_ can be evaluated with the following equation [22],

(1) $$\Gamma (\phi )\;=\frac{\sum_{n=1}^{N}F_{n}\left(\phi \right)Y_{n}}{\sqrt{\sum_{n=1}^{N}F_{n}^{2}\left(\phi \right)}\sqrt{\sum_{n=1}^{N}Y_{n}^{2}}},\;N=4$$

**Figure 14:**
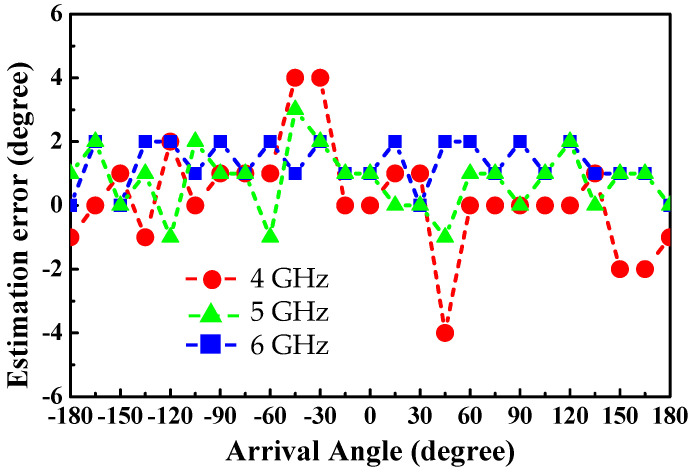
Measured estimation error of angle-of-arrival system, using Ant D at 4, 5, and 6 GHz.

**Figure 15:**
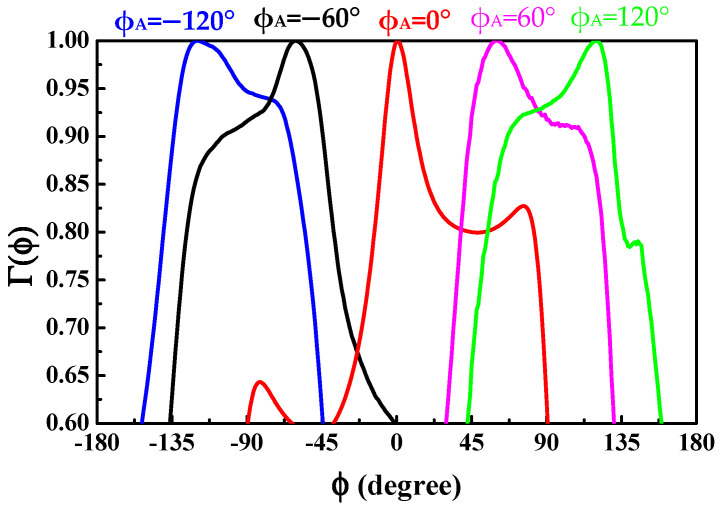
Variations in Γ(ϕ) against ϕ measured at 5 mm.

The direction of the transmitter can be estimated by finding the maximum of Γ(*ϕ*), and the corresponding *ϕ* is the angle of arrival; that is, *ϕ*_A_. We note that Equation (1) defines the correlation coefficient as the normalized dot product between the observation vector, *Y_n_*, and the reference pattern, *F*_n_(ϕ). Therefore, Γ(ϕ) represents the squared projection of the observed vector onto the reference space, where a maximum value indicates the most probable direction of arrival. For the proposed AoA estimation method, it is based solely on amplitude information without requiring phase measurements or complex-valued signal processing. Figure 14 presents the measured data when the signal is transmitted at different angles of the whole horizontal plane. From the results, it can be observed that the maximum errors are 4°, 3°, and 2° when Ant D is operated at 4, 5, and 6 GHz, respectively. Moreover, the results also indicate that the estimation mean error is less than 1.5° over the whole horizontal plane when Ant D is applied in the angle-of-arrival system, operating in the frequency band from 4 to 6 GHz. For the cases of *ϕ*_A_ = 0°, ±60°, and ±120°, the variations in Γ(ϕ) against ϕ are presented in Figure 15. For each distinct *ϕ*_A_, the Γ(ϕ) curve exhibits a sharp peak at its corresponding angle, validating that the correlation algorithm can robustly distinguish different incident directions within the full 360° horizontal plane based on the switched-beam amplitude response.

According to the measured results in Figure 12, the half-power beamwidth decreases from approximately 68° to 54° as the frequency increases from 4 to 6 GHz. A narrower beamwidth provides higher angular resolution and reduces the overlap between adjacent radiation patterns, thereby enhancing the distinguishability of received signal profiles corresponding to different directions. Consequently, the correlation-based estimation process can more precisely identify the angle of arrival. Overall, the obtained results demonstrate that the proposed antenna maintains stable radiation characteristics and reliable directional discrimination over a wide bandwidth. Despite providing full 360° angular coverage, the system achieves reliable wide-angle AoA estimation with low hardware complexity, making it well suited for wideband sensing and integrated sensing and communication applications.

## 6. Conclusions

A design for beam-steerable antennas has been presented. The design is based on a circular array comprising eight elements with wideband operation. To improve the gain of the switched beam, a pair of metallic plates are introduced. The proposed design has a simple structure, and the total size is 1.3 λ_0_ × 1.3 λ_0_ × 0.85 λ_0_. An antenna prototype integrated with the related circuits is constructed to carry out electrical beam scanning. The experimental results demonstrate that the antenna can deliver four symmetrical beams in the whole horizontal plane within an impedance bandwidth of 50%, and the maximum gain of the beams can reach up to 8.3 dBi; moreover, a side lobe level smaller than −10 dB is also obtained. With the above performances, the proposed reconfigurable antenna has been successfully applied to AoA systems.

## Figures and Tables

**Table 1:** Comparisons between the proposed and reported antennas with steerable beams: BW, 10 dB return loss bandwidth; Gain, maximum gain within BW. Back lobes are considered in SLLs.

Ref.	BW (%)	Gain (dBi)	SLL (dB)	Size (λ_0_ × λ_0_ × λ_0_)	AoA Capability
[8]	8	4.9	−8	0.36 × 0.36 × 0.1	No
[9]	33.6	4.1	−10	0.61 × 0.61 × 0.007	No
[10]	12	7.3	−7	1.8 × 1.8 × 0.03	No
[14]	71	6.4	−3	0.72 × 0.72 × 0.012	No
[16]	8.5	8.6	−10	0.71 × 0.71 × 2	No
[17]	18.2	8.2	−5	1.67 × 1.67 × 1.03	No
[18]	14.5	10	−8	2.5 × 2.5 × 0.5	No
[19]	26.2	9.7	−10	3.1 × 3.1 × 1	No
This work	50	8.3	−10	1.3 × 1.3 × 0.85	Yes (<1.5°)

## Data Availability

The data presented in this study are available on request from the corresponding author.
